# Consumption of Green Coffee Reduces Blood Pressure and Body Composition by Influencing 11*β*-HSD1 Enzyme Activity in Healthy Individuals: A Pilot Crossover Study Using Green and Black Coffee

**DOI:** 10.1155/2014/482704

**Published:** 2014-07-16

**Authors:** R. Revuelta-Iniesta, E. A. S. Al-Dujaili

**Affiliations:** Department of Dietetics, Nutrition and Biological Sciences, Queen Margaret University, Queen Margaret University Drive, Musselburgh, East Lothian, Edinburgh EH21 6UU, UK

## Abstract

Dietary polyphenols may have a protective role against the development of CVD. Thus, we aimed to investigate the effects of green coffee (GC), rich in chlorogenic acid, and black coffee (BC) on cardiovascular markers. A randomised pilot crossover study was performed on healthy subjects who consumed both coffees for 2 weeks. We measured anthropometry, blood pressure, and arterial elasticity after each intervention and collected urine samples to monitor antioxidant capacity. Free cortisol and cortisone levels were obtained from urine and analysed by specific ELISA methods. Systolic blood pressure (*P* = 0.018) and arterial elasticity (*P* = 0.001) were significantly reduced after GC. BMI (*P* = 0.04 for BC; *P* = 0.01 for GC) and abdominal fat (*P* = 0.01 for BC; *P* = 0.009 for GC) were also significantly reduced with no changes in energy intake. Urinary free cortisol was significantly reduced from 125.6 ± 85.9 nmol/day to 76.0 ± 54.9 nmol/day following GC and increased to 132.1 ± 89.1 nmol/day after BC. Urinary free cortisone increased by 18% following BC and 9% following GC (nonsignificant). Cortisol/cortisone ratio (indicating 11*β*-HSD1 activity) was reduced after GC (from 3.5 ± 1.9 to 1.7 ± 1.04, *P* = 0.002). This suggests that GC can play a role in reducing cardiovascular risk factors. Further research including hypertensive and overweight individuals will now be justified to clarify whether GC could have a therapeutic role in CVD.

## 1. Introduction

Cardiovascular disease (CVD) is the leading cause of mortality and morbidity worldwide and there are several main risk factors for its development which include dyslipidaemia, smoking, diabetes, hypertension, and obesity [1]. Elevated plasma glucocorticoid levels lead to an increase in abdominal obesity and associated metabolic complications such as type 2 diabetes [2]. Cortisol is a steroid hormone (glucocorticoid) secreted in response to stress and low blood cortisol levels to restore homeostasis in the body [3]. In acute stress catecholamines mobilise glucose and fatty acids to provide the muscles and organs with enough energy for action and, in this instance, glucocorticoids protect the body against overreactions to stress. However, in chronic stress of food and fluid deprivation, glucocorticoids stimulate gluconeogenesis and increase the mobilisation of free fatty acids from subcutaneous and adipose tissues and amino acids from skeletal muscle, which in the long term contribute to central obesity. An association between cortisol and hypertension has also been shown [4]. There are several mechanisms proposed by which cortisol is thought to increase blood pressure (BP): (i) it decreases the production and bioavailability of nitric oxide (NO), which is a vasodilator [4]; (ii) it also increases the responsiveness to vasoconstrictors such as the endothelin (ET) system, which play an active role in the pathogenesis of atherosclerosis [3], and stimulates sodium reabsorption in the kidneys to preserve water [5].

Some authors reported that consumption of antioxidant-rich foods and beverages has a protective role against the development of risk factors for CVD [6]. Coffee, especially green coffee (GC), is a known rich source of chlorogenic acid (CGA), which is a type of polyphenol, and caffeine. CGA is a phenolic compound with antioxidant activity [6] and high bioavailability [7–9]. CGA may have antihypertensive and weight reducing effects [10–12]. Both the blood pressure and weight reducing effects have been attributed to the inhibition of 11*β*-hydroxysteroid dehydrogenase type 1 (11*β*-HSD1) present in the adipose tissue and the liver and involved in the conversion of the hormonally inactive cortisone (11-dehydrocorticosterone in rodents) into active cortisol (corticosterone in rodents) [10]. The weight loss effect of CGA has also been associated with a reduction in the absorption of glucose [10–12].

The composition of black coffee (BC) differs from that of the green coffee due to the roasting process. As a result their effects might also differ. BC may raise blood pressure, identifying caffeine as the main BP-raising agent [13]. This effect has been attributed to the stimulation of the sympathetic nervous system, which in turn increases the rate and force of cardiac contractility, leading to a temporary increase in blood pressure [13]. In contrast, other studies suggest that coffee might lower blood pressure due to a development of tolerance to caffeine in regular coffee drinkers [14, 15]. Habitual coffee consumption increases resting energy expenditure by about 100 kcal/day, which in the long term may lead to a significant weight loss [16, 17]. Moreover, regular intake of coffee encourages the excretion of Na and water and the retention of K in the nephron [18], which may reduce BP.

Taking into consideration the health benefits attributed to CGA from green coffee and the inconsistent findings regarding the effects of black coffee, we aimed to (i) investigate the effects of green coffee and black coffee in comparison to baseline and (ii) compare the effects of green coffee and black coffee on cardiovascular markers, including physiological parameters (blood pressure and arterial elasticity), anthropometry and body composition (weight, waist circumference, and abdominal fat), and glucocorticoids levels in healthy volunteers.

## 2. Subjects and Methods

### 2.1. Study Population

The study protocol was approved by the Queen Margaret University's (QMU's) Research Ethics Committee in July 2011. The study was advertised in the QMU website and interested volunteers were given detailed information about the study. Eligibility criterion was assessed using a health status questionnaire, which also asked about physical activity habits. We included healthy subjects with a BMI between 18 and 30 kg/m^2^ and no history of CVD, including stroke, hypertension (WHO/ISH: SBP ≥ 150 mmHg and DBP ≥ 90 mmHg), coronary heart disease, and diabetes mellitus. Those with a kidney condition or gastrointestinal pathologies were all excluded. Smokers were excluded too [8, 19]. All data collected from the volunteers was kept anonymous and all measurements took place in the QMU clinic room.

### 2.2. Study Design

We conducted a pilot study. Our design was randomised and crossover, in which a repeated measure design was also used. The randomisation was performed using a software program (Clinstat, MS = DOS) by Martin Bland [20], which is suitable for small scale trials. A printed list of random allocations was obtained. Subjects were then allocated into 2 groups; some were given the BC first and others started on the GC. All measurements were taken on three occasions: at baseline, after the black coffee, and after the green coffee intervention. A week washout period was allowed in between interventions to avoid a carryover effect [21]. Volunteers completed a 2-day diet diary on three occasions (at baseline and during each intervention) including a weekend and week day. The volunteers were asked to follow their usual eating and physical activity patterns. Questions about physical activity were asked at the start of the study (baseline) and at the end of each intervention using the same health status questionnaire. The diet diaries were analysed using Windiet 2005. The intervention period was 14 days for each arm of the study.

### 2.3. Coffee

The GC used in this project was Ethiopian Harrar 4 (100% Arabica) and the BC was Sainsbury's Original Blend Cafetière Coffee. The BC was a blend of Brazilian, Colombian, Mexican, Nicaraguan, Peruvian, and Rwandan beans. Subjects were asked to have 40 g of GC and BC per day distributed throughout the day into four cups of coffee. This ensured that high plasma coffee antioxidant concentrations were maintained over a period of time, allowing effects to take place and the body to develop tolerance to caffeine, which can take 2-3 days [13]. Although the amount of coffee given was the same, GC contains 9% of water, which is higher than the water content of BC, as this evaporates in the roasting process [12]. The GC beans were grounded to powder using an electric coffee grinder. Instructions on how to make the coffee were provided and the Italian cafetière, the French cafetière, or the filter coffee machine was used to prepare the coffee drinks.

### 2.4. Anthropometry and Body Composition Measurements

Height (m) was measured using a leister-height scale and weight (Kg) with a digital scale “Salter” model 9106. Measurements were obtained using standard protocols and the same equipment was employed every time to avoid inaccuracies between different scales. Body mass index (BMI) was then calculated using the following standard equation: BMI (Kg/m^2^) = (weight Kg/(height m)^2^). Upper percentage body fat was measured using a Body Fat Monitor BF306 (Omron) and waist circumference (cm) was taken following WHO guidelines (2008) [22].

### 2.5. Blood Pressure and Arterial Elasticity Measurements

A digital sphygmomanometer (Omron M5-I) was used to measure BP while the subjects lied down comfortably in a 30-degree position and were allowed to relax for 10 minutes in a quiet room to avoid “white coat” hypertension. The pulse wave velocity (PWV) was measured using a noninvasive devise, Vicorder (Dell) equipment. To ensure reliability of results; values were measured three times and mean was calculated.

Volunteers collected urine over 24-hour period on three occasions: at baseline and at day 14 of each intervention. All samples were consequently weighed and stored in sample tubes in the freezer at −20°C. The 24-hour urine collection was evaluated in two ways: (i) by observing the urine output at each stage of the intervention and then comparing the three volumes obtained at the end and (ii) by monitoring the creatinine of three random samples. The total polyphenol content of these samples was analysed using Folin and Ciocalteau's method [23]. The method used to analyse the reducing (antioxidant) capacity was Ferric ion reducing antioxidant power (FRAP) [24]. The polyphenol concentration and antioxidant capacity of the different coffee preparations were analysed using the same methods and absorbance was measured using Sperk and Burke SB analytical spectrophotometer (He*λ*ios *α*). Urinary cortisol and cortisone levels were analysed by using indirect competitive sensitive and specific ELISA methods according to the protocol devised by [25].

### 2.6. Statistical Analysis

All statistics were performed using SPSS statistics version 17.0 and variables were first tested for normal distribution. Nonparametric variables were transformed to parametric using the log transformation option and then analysed using a 2-tail paired *t*-test. Results are expressed as mean ± standard error of mean (SEM). *P* values of ≤0.05 were considered significant.

## 3. Results

### 3.1. Subjects Characteristics

Twenty healthy volunteers participated in this study (13 females and 7 males) (Figure 2). One volunteer dropped out and did not give a reason and another completed only part of the study as the urine collection was inconvenient. Therefore, 18 subjects completed the whole intervention. All volunteers apart from two were moderate regular coffee drinkers consuming 2–4 cups a day: 35% of the research project population reported to be sedentary, while the other 65% did regular exercise of at least 30-minute walk a day.

### 3.2. Diet and Physical Activity

The phenolic and antioxidant concentrations present in 10 g/100 mL of green coffee Ethiopian Harrar 4 (GC) and 10 g/100 mL of Sainsbury's Original Blend Cafetière coffee (BC) are presented in Table 1. Volunteers used a mixture of methods during the intervention.

Volunteers were asked at the end of each intervention to report any dosage missed and compliance was calculated accordingly. Subjects had an estimated mean compliance of 94.5% for the GC, with only one subject missing a day dosage of GC. While for the BC there was 92% compliance with a maximum dosage missed of one and a half days.

As it can be seen from Table 2, the total energy intake and sodium intake reported from the diet diaries did not significantly differ between baseline and each intervention. Finally, all volunteers reported in the health status questionnaire having maintained their physical activity throughout the whole study.

### 3.3. Effect on Physiological Markers

GC significantly reduced systolic BP (*P* = 0.02) by mean ± SEM 2.65 ± 1.37 mmHg compared to baseline. BC reduced systolic BP by 2.23 ± 1.44 mmHg, but this was not significant. No statistical significant difference was found when the two interventions were compared against each other. Diastolic BP was significantly reduced after both interventions: 2.9 ± 1.05 mmHg after BC and 3.1 ± 0.81 after GC, and the comparison between the two interventions was not significant (Table 2).

Finally PWV mean values also decreased by 0.20 ± 0.12 m/second following BC intake and by 0.26 ± 0.12 m/second after GC, only significant after GC (*P* = 0.001). No significant difference was obtained between BC and GC (*P* = 0.49) (Table 3).

### 3.4. Effect on Biochemical Markers

No significant difference was seen in the antioxidants' capacity and polyphenol concentration from the urine samples. Cortisol levels decreased significantly by 39% after the GC intervention and increased by 5% after BC (there was a significant difference between interventions). While cortisone levels were 18% and 9% higher following BC and GC intervention, respectively, this was not significant. BC and GC significantly reduced 24-hour urinary free cortisol/cortisone ratio and there was a significant difference when the two interventions were compared (*P* = 0.02; Figures 3 and 4; Table 3).

### 3.5. Effect on Anthropometrical Measurements

At baseline, the participant's weight and BMI were 70.52 ± 15.89 kg and 24.23 ± 4.6 kg/m^2^, respectively. This was only significantly reduced after GC (*P* = 0.01 for both parameters) to 69.13 ± 15.9 kg and 23.95 ± 4.78 kg/m^2^, respectively.

Waist circumference decreased by an average of 0.89 cm after the BC intervention and 1.74 cm after the GC intake compared to baseline. Abdominal fat decreased by an average of 1.44% (BC) and 1.75% (GC). Both were significantly reduced after each intervention (WC: *P* = 0.04 (BC) and *P* = 0.009 (GC); abdominal fat: *P* = 0.01 (BC) and *P* = 0.015 (GC)). No significant difference was seen between BC and GC in either of the two anthropometrical parameters (*P* = 0.9) (see Table 4). There were no significant differences observed in energy and sodium intake at baseline and at the end of both interventions.

### 3.6. Adverse Events

One volunteer reported feeling “a bit dizzy” and “nauseous” immediately after ingesting the GC and another reported an increase in libido. The first one was linked to the substantial reduction in BP that volunteer had. No other adverse effects were reported or noted during or after the interventions.

## 4. Discussion

The results from our pilot study showed that consumption of green coffee significantly improved arterial elasticity and reduced systolic and diastolic blood pressure in the healthy cohort included in the study. Black coffee also lowered systolic and diastolic blood pressure, but only the latter was statistically significant. Additionally, the results demonstrated that consumption of both coffees for two weeks, during which subjects did not modify their diet and physical activity, led to a significant reduction in body weight, waist circumference, and % abdominal fat. There was a significant decrease in the stress hormone urinary free cortisol levels only after GC, and urinary free cortisone (inactive form) levels increased slightly after both interventions. The ratio cortisol/cortisone was significantly reduced following both BC and GC consumption indicating that coffee intake may inhibit 11*β*HSD1 enzyme activity. However, there was no significant change in either the total urinary polyphenol concentration or the antioxidant capacity after interventions.

### 4.1. Blood Pressure and Arterial Elasticity

The guidelines issued by NICE (2006) stated that the risk associated with increasing BP is continuing [1]. Each 2 mmHg rise in systolic BP is associated with a 7% increased risk of mortality from ischaemic heart disease and 10% from stroke [1]. The blood pressure lowering effects of GC reported in this study of 2.65 ± 1.37 mmHg for systolic BP and 3.10 ± 0.81 mmHg for diastolic BP in normotensive individuals may also reduce blood pressure in hypertensive individuals. The effects reported in our study are corroborated by others [10, 25]. Kozuma et al. [10] demonstrated a reduction in systolic BP of 5 mmHg and in diastolic BP of 4.7 mmHg in mildly hypertensive individuals after the ingestion of green coffee bean extract (GCBE) over a period of 28 days and a dose of 93 mg/day. Almoosawi et al. [26] meanwhile found a significant reduction in systolic BP after testing the effects of GCBE for 2 weeks in 13 overweight volunteers. In contrast, Watanabe et al. [27] did not find any significant changes in a 4-month study performed using normotensive male individuals, even though an average reduction of 4.6 mmHg (SBP) and 3.2 mmHg (DBP) was observed at the end of the intervention period, which might have been due to the small sample size.

Previous research showed an association between habitual coffee intake and an increase in arterial elasticity [9]. Habitual coffee consumption had an inverse association with surface leukocyte adhesion molecules (E-selectin) and C-reactive protein in healthy and diabetic women, which in turn improved arterial stiffness. This was attributed to the strong antioxidant capacity present in coffee [9]. Our study only found a significant reduction in PWV following GC intervention. Yet a significant difference in neither the polyphenol concentration nor the antioxidant capacity was obtained. This raises the question of why there was an improvement in arterial elasticity without changes in the antioxidant capacity and why the BP lowering effect from GC was more potent than that of BC.

**Figure 1:**
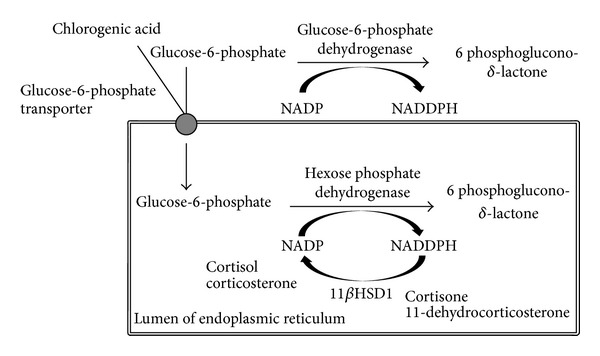
Inhibition of glucose-6-phosphate by chlorogenic acid. CGA is involved in blocking the uptake of glucose-6-phosphate by inhibiting glucose-6-phosphate translocase. This prevents the enzyme glucose-6-phosphate dehydrogenase from providing NADPH as a cofactor for the reductase activity and so the conversion of cortisone to cortisol is inhibited. NADPH: nicotinamide adenine dinucleotide phosphate. Adapted from a Ph.D. thesis by Al Moosawi, Queen Margaret University, 2010 [42].

**Figure 2:**
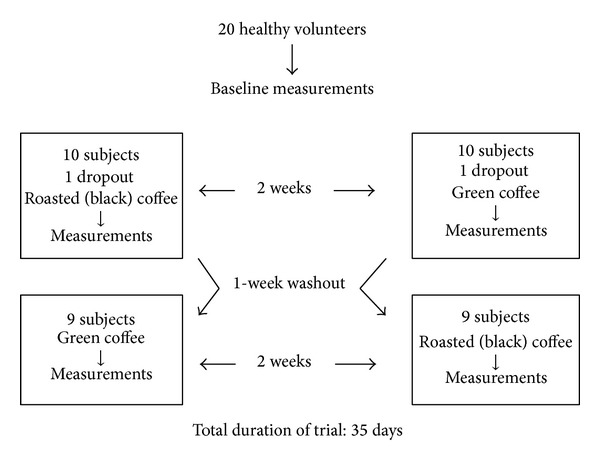
Flow diagram of our crossover study protocol and sample size.

**Figure 3:**
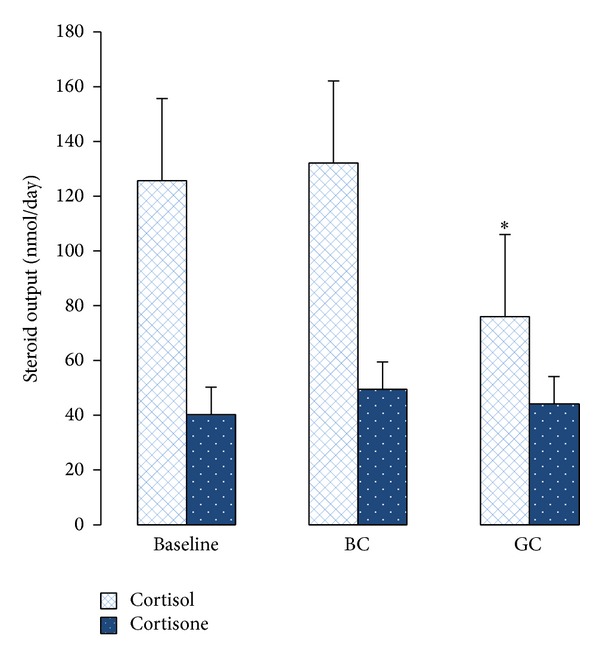
Urinary free cortisol and cortisone levels (nmole/24 hours) obtained before and after consumption of green and black coffees. Results are expressed as mean ± SEM; *P = 0.01 after Wilcoxon test.

**Figure 4:**
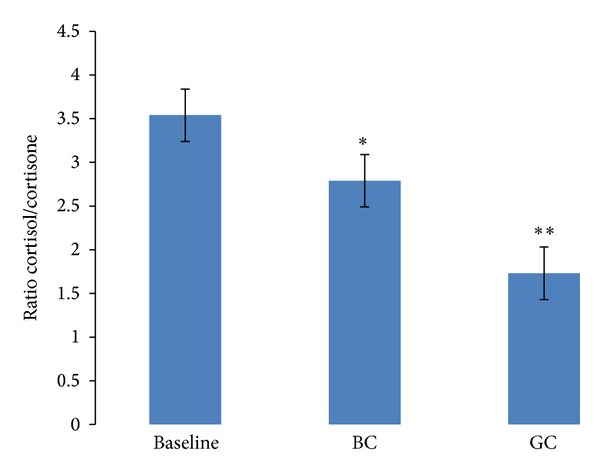
Urinary free cortisol/cortisone ratio levels obtained before and after consumption of black and green coffee. Results expressed as 24 h mean ± SEM. There was a significant reduction in urinary free cortisol/cortisone ratio after consumption of black coffee (*P = 0.04) and green coffee (**P = 0.003).

It is well established that high cortisol levels contribute to hypertension by overstimulation of the hypothalamic adrenal axis and sympathetic nervous system [28] and those individuals appear to have higher levels of urinary free cortisol and catecholamines metabolites. Our results illustrate a decrease in 24-hour urinary free cortisol, the cortisol/cortisone ratio, and the BP after GC. Catecholamine's metabolites were not analysed. Thus, our hypothesis is that chlorogenic acid present mainly in GC has an inhibitory effect on the enzyme 11-*β*HSD1 (Figure 1), whilst the active BC compound produced during the roasting process has a stimulant effect on 11-*β*HSD2 [29]. In addition, the inhibitory effect on the enzyme 11*β*-HSD1 is 7–10-fold higher than the 11*β*-HSD2, which may explain the higher effect of GC on the urinary free cortisol/cortisone ratio. Secondly, cortisol might reduce the production and bioavailability of vasodilators such as nitric oxide [30]. Thus, a reduction of cortisol levels may have led to an improvement in arterial elasticity and BP.

A reduction in both PWV and BP is not entirely surprising since they are intrinsically related. The pulse wave generated by the contraction of the left ventricle travels towards the periphery where a part of it is reflected back [19]. In elastic arteries, this wave returns travelling slowly and arrives during diastole facilitating then coronary perfusion [19]. With arterial stiffness this wave arrives early, before the aortic valve closes, increasing therefore SBP or the pressure that the left ventricle needs to propel the blood to reach the peripheral vessels [19].

Results concerning the effects of black coffee intake on BP and PWV are still inconsistent. Our study showed a significant reduction in diastolic BP only. Although, both systolic BP and PWV were reduced, neither was significant. This may be attributed to the following: (i) the characteristics of our volunteers, as 18 out 20 were regular coffee consumers [9, 14, 31–35], findings not seen in nonregular coffee consumers [9, 14]; (ii) the diuretic properties of coffee [36, 37] brought by the inhibition of plasma renin activity [36, 37] and the high concentrations of potassium present in coffee, also seen in the significant increase in potassium intake of our volunteers (Table 3) [14]. Consequently, the presence of both CGA in green coffee and CGA metabolites, as well as the high concentrations of potassium [14], could be the agents that neutralise the effects of caffeine and have the lowering effects of BP seen in our cohort.

### 4.2. Weight, Waist Circumference, and Abdominal Fat

This study has shown that BMI fell following the two interventions; however, this was only significant following GC intake. Both coffees significantly reduced abdominal fat despite energy intake and physical activity remaining the same throughout the intervention.

The effect of GC in reducing body weight is supported by others [21]. The author of [21] reported a significant weight loss of 5.4 kg after 12 weeks of Coffee Slender intake only; however, the sample size was also small [15]. In contrast to our findings, a prospective cohort study involving subjects aged 32–88 years showed an association between BC consumption and weight reduction [38]. The weight lowering effect of green coffee has been attributed to the effects of CGA [21, 39]. CGA can reduce and slow down the uptake of glucose in the small intestine and caffeine increases resting energy expenditure [21, 39]. Perhaps the difference between the two interventions is explained by the fact that the concentrations of both CGA and caffeine are different, being both higher in GC. CGA slows down the absorption of glucose in the small intestine and it was thought to inhibit fat accumulation in the abdominal area and caffeine is known to increase lipolysis and total energy expenditure. Furthermore, CGA is known to inhibit 11*β*-HSD 1 and 2 activity. This could also be the cause of the reduction in urinary free cortisol levels seen in this study, which in turn may have led to the reduction in BP and the improvement in arterial elasticity.

Finally, the waist circumference and abdominal obesity reduction shown in this study, especially following GC, supports the hypothesis that a decrease in visceral adiposity occurred as a result of the inhibition of 11*β*-HSD1 expression by CGA that was evident by the reduction of urinary free cortisol/cortisone ratio after both interventions.

There are a number of limitations in this study. We acknowledge that the sample size of our study is relatively small and that the age range is [25–41], which makes the extrapolation to the general or specific population difficult; nevertheless, all volunteers were healthy and further research will now be justified to investigate these findings using a larger sample and more specific populations. Accuracy of the Body Fat Monitor BF306 (Omron) has been argued as the reported error of measurement is 4.1% which might have led to errors in assessing the percentage (%) abdominal adiposity. However, the waist circumference was also measured, and both measurements were in agreement, which allowed the validation of the results obtained in this study. Although, the activity of 11*β*-HSD1 and 11*β*-HSD2 were not directly measured, 24-hour urinary collection was obtained from the volunteers and compliance was evaluated, allowing the analysis of both cortisol and cortisone levels and the consequent calculation of their secretion ratio, which in turn can assess the activity of the enzyme. Additionally, the volunteers were asked to follow similar physical activity and eating patterns during the two interventions, but diet diaries were only recorded for two days on three occasions and might not represent the intake of the whole intervention. Also, we used questionnaires to assess whether volunteers maintained their usual physical activity patterns, which might have not been a true representation. Finally, our study was not blinded, which may have led to bias, and the possibility of significant findings by chance may have occurred due to both multiple testing and changes that occur over time when participating in trial.

## 5. Conclusion

This pilot study proposes that coffee intake, especially GC, might have a role to play in the reduction of cardiovascular risk factors and perhaps CVD in the long term. Further research (including blinded clinical trials, although this might be difficult) using a larger sample of hypertensive and overweight individuals, as well as longer intervention period, is warranted to clarify whether GC could have a preventative or a therapeutic role in the fight against CVD.

## Figures and Tables

**Table 1:** Concentration of total polyphenols and antioxidant capacity determined in GC and BC as compared by the three methods of coffee preparation.

10 g BC in 100 mL water	Filter	Italian	French
10 g GC in 100 mL water	Method	Cafetière	Cafetière
FRAP mMFeII (BC)	12.8	14.1	19.9
FRAP mMFeII (GC)	11.7	16.6	10.3
Polyphenols mg GAE (BC)	1451	1496	2475
Polyphenols mg GAE (GC)	1360	2052	972

**Table 2:** Effect of black coffee (BC) and green coffee (GC) on physiological markers, total polyphenols, and antioxidant capacity (FRAP) in 24-hour urine samples (mean ± SEM).

Study population	Baseline	BC	Baseline versus BC (95% CI)	GC	Baseline versus GC (95% CI)
SBP (mmHg)	116.9 ± 2.4	114.5 ± 2.5	−0.81 to 5.27	113.5 ± 3.1^1^	1.2 to 5.5
DBP (mmHg)	74.0 ± 1.7	71.2 ± 1.7^2^	0.77 to 5.20	71.2 ± 2.2^3^	1.4 to 4.8
PWV (m/sec)	6.63 ± 0.20	6.40 ± 0.20	−0.04 to 0.44	6.29 ± 0.30^4^	0.2 to 0.5
Total polyphenols (mgGAE/day)	523 ± 33	597 ± 44	−175 to 27	547.78 ± 36.4	−156 to 106
FRAP (mMFeII/day)	7.22 ± 3.38	8.85 ± 4.53	−3.5 to 0.4	7.21 ± 3.33	−1.7 to 2.5
Energy intake (kcal)	1807 ± 29	1903 ± 31	−328 to 164	1853 ± 29	−245 to 113
Sodium intake (mg)	2571 ± 277	2609 ± 291	−429 to 675	2490 ± 277	−268 to 719
Potassium intake (mg)	2681 ± 190	3310 ± 238	−869 to −118^2^	3161 ± 276	−933 to 233

BC: black coffee; GC: green coffee; SBP: systolic blood pressure, DBP: diastolic blood pressure; PWV: pulse wave velocity; CI: 95% confidence interval; significance expressed as *P* ≤ 0.05; 2-tail paired *t*-test; ^1^baseline versus GC, *P* = 0.02; ^2^baseline versus BC, *P* = 0.01; ^3^baseline versus GC, *P* = 0.001; ^4^baseline versus GC, *P* = 0.001.

**Table 3:** Comparison of results obtained (mean ± SEM) after 14 days of green coffee and black coffee intervention.

Study population	Black coffee	Green coffee	Difference	95% CI	*P*
BMI (kg/m^2^)	23.9 ± 1.1	23.9 ± 1.1	0.0	−0.21 to 0.22	*0.9 *
Waist Circumference (cm)	79.2 ± 3.3	79.1 ± 3.4	0.1	−0.53 to 0.67	*0.8 *
Abdominal fat (%)	28.9 ± 1.9	28.4 ± 2.0	0.5	−0.13 to 1.18	*0.1 *
SBP mmHg	114.5 ± 11.2	113.5 ± 13.3	1.0	−11.43 to 7.76	*0.6 *
DBP mmHg	71.2 ± 7.6	71.2 ± 9.9	0.0	−9.32 to 5.75	*0.5 *
PWV m/sec	6.4 ± 0.9	6.3 ± 1.2	0.1	−0.55 to 0.33	*0.5 *
Polyphenol (mgGAE/day)	597 ± 241	548 ± 211	49.0	−55.11 to 153.95	*0.3 *
Antioxidant (mMFeII/day)	8.8 ± 4.5	7.2 ± 3.3	1.6	−0.30 to 4.14	*0.08 *
Energy intake (kcal)	1880 ± 147	1863 ± 128	17.0	−256 to 291	*0.9 *
Sodium intake (mg)	2609 ± 291	2490 ± 277	119.0	−228 to 467	*0.5 *
Potassium intake (mg)	3310 ± 238	3161 ± 276	149.0	−304 to 602	*0.5 *
Urinary free F (nmol/day)∗	114.5 ± 331.7	68.4 ± 64.6	46.1	12.4 to 93.7	*0.01 *
Urinary free E (nmol/day)∗	40.9 ± 36.6	47.7 ± 26.5	−6.8	−6.26 to 15.8	*0.5 *
Urinary F : E ratio (nmol/day)	2.55 ± 2.58	1.53 ± 1.42	1.02	0.14 to 1.78	*0.02 *

95% CI: 95% confidence interval; *P* values are from paired *t*-test. ∗Variables normalised using the log⁡10 (SPSS 17.0). F: cortisol; E: cortisone.

**Table 4:** Characteristics of population after 14 days of black coffee and green coffee intake expressed as mean ± SEM.

Study population	Baseline	BC	Baseline versus BC (95% CI)	GC	Baseline versus GC (95% CI)
BMI (kg/m^2^)	24.3 ± 1.0	23.9 ± 1.1^1^	0.01 to 0.40	23.9 ± 1.1^2^	0.05 to 0.30
Weight (Kg)	70.8 ± 3.5	69.3 ± 3.5	−0.10 to 0.80	69.1 ± 3.5^3^	0.20 to 0.90
Waist circumference (cm)	80.8 ± 3.2	79.2 ± 3.3^4^	0.08 to 1.80	79.1 ± 3.4^5^	0.30 to 1.80
Abdominal fat (%)	30.1 ± 1.9	28.9 ± 2.0^6^	0.20 to 2.80	28.4 ± 2.0^7^	0.39 to 3.30

BC: black coffee; GC: green coffee; BMI: body mass index; 95% CI: 95% confidence intervals; 2-tail paired *t*-test, significance expressed as *P* ≤ 0.05; ^1^B versus BC, *P* = 0.04; ^2^B versus GC, *P* = 0.01; ^3^B versus GC, *P* = 0.006; ^4^B versus BC, *P* = 0.0.03; ^5^B versus GC, *P* = 0.009; ^6^B versus BC, *P* = 0.02; ^7^B versus GC, *P* = 0.01.

## Abbreviations

AOX: Antioxidant
BC: Black coffee
BP: Blood pressure
CGA: Chlorogenic acid
CVD: Cardiovascular disease
DBP: Diastolic blood pressure
ELISA: Enzyme-linked immunosorbent assay
E: Cortisone
F: Cortisol
FRAP: Ferric antioxidant power
GAE: Gallic acid equivalent
GC: Green coffee
11 *β*-HSD1: 11 *β*-Hydroxysteroid dehydrogenase type 1
NO: Nitric oxide
PWV: Pulse wave velocity
ROS: Reactive oxygen species
SBP: Systolic blood pressure.
